# Association of Preoperative Linear MRI Measures with Domain-Specific Cognitive Change After Subthalamic Nucleus Deep Brain Stimulation in Parkinson’s Disease

**DOI:** 10.3390/jcm14238414

**Published:** 2025-11-27

**Authors:** Stanisław Szlufik, Karolina Szałata, Patryk Romaniuk, Karolina Duszyńska-Wąs, Magdalena Karolak, Agnieszka Drzewińska, Tomasz Mandat, Mirosław Ząbek, Tomasz Pasterski, Mikołaj Raźniak, Dariusz Koziorowski

**Affiliations:** 1 Department of Neurology, Faculty of Health Sciences, Medical University of Warsaw, 02-091 Warsaw, Poland; 2 Department of Neurosurgery, Maria Sklodowska-Curie National Research Institute of Oncology, 02-781 Warsaw, Poland; 3 Department of Neurosurgery, 1st Military Teaching Hospital in Lublin–Branch in Ełk, 19-300 Ełk, Poland; 4 Students Scientific Group, Department of Neurology, Faculty of Health Sciences, Medical University of Warsaw, 02-091 Warsaw, Poland; 5 Department of Neurosurgery, Mazovian Bródno Hospital, 02-507 Warsaw, Poland; 6 Department of Neurosurgery, Center of Postgraduate Medical Education, 00-416 Warsaw, Poland

**Keywords:** Parkinson’s disease, deep brain stimulation, subthalamic nucleus, cognitive decline, MRI morphometry, verbal fluency, hippocampus, amygdala, lateral ventricles, neuropsychology

## Abstract

**Background/Objectives**: Deep brain stimulation of the subthalamic nucleus (STN-DBS) is an effective treatment for motor symptoms in Parkinson’s disease (PD), but concerns remain regarding its impact on cognitive function. Identifying neuroanatomical predictors of postoperative cognitive decline could improve patient selection and outcomes. This study aims to investigate the relationship between preoperative brain morphology and postoperative neuropsychological outcomes in PD patients undergoing bilateral STN-DBS. **Methods**: Thirty-eight PD patients underwent standardized neuropsychological testing and preoperative MRI before and 3–24 months after STN-DBS. Manual MRI morphometric measurements were obtained for 42 cortical, subcortical, and ventricular parameters. Changes in cognitive domains—including executive function, memory, language, visuospatial abilities, attention, and global cognition—were analyzed, and correlations between structural metrics and cognitive changes were assessed using Spearman’s coefficients. **Results**: Significant postoperative declines occurred selectively in language functions: verbal fluency (phonemic and semantic, d = −0.49 to −0.84) and confrontation naming (d = −0.47). Memory, executive functions, attention, and global cognition remained preserved. Enlarged lateral ventricles were consistently associated with poorer outcomes across multiple domains, while increased left precentral gyrus width correlated with executive and memory decline. Additionally, smaller midbrain and cingulate gyrus width were associated with greater executive impairment. **Conclusions**: STN-DBS in PD is associated with selective postoperative cognitive changes, most prominently in verbal fluency. Simple preoperative MRI morphometric measures, including ventricular size, limbic structure volumes, and specific cortical parameters, may serve as clinically feasible predictors of cognitive risk. Incorporating such measures into preoperative assessments could enhance patient selection, counseling, and individualized surgical planning.

## 1. Introduction

Deep brain stimulation (DBS) is an invasive neurosurgical intervention involving the implantation of electrodes into deep brain structures, such as the Subthalamic Nucleus (STN). It is utilized in the management of Parkinson’s disease (PD), particularly for patients whose primary symptom is tremor. This procedure can mitigate motor symptoms and enhance patients’ quality of life. However, in recent years, there has been increasing concern regarding its impact on the cognitive function of individuals with PD. Studies indicate that while DBS may yield positive therapeutic outcomes, it also poses a long-term risk of cognitive decline [1]. Cognitive status is a critical determinant of eligibility for DBS, and cognitive decline represents a significant adverse effect. Consequently, it is imperative to identify markers that could serve as potential predictors of cognitive deterioration. Depending on the anticipated risk of cognitive decline, alternative therapies, such as DBS in the globus pallidus internus (DBS Gpi), may be considered [2]. Research further suggests that DBS does not result in general cognitive impairment but rather in a selective decline in specific cognitive functions [3]. Therefore, it is essential to monitor changes in various cognitive functions through diverse neuropsychological assessments, and it appears prudent to incorporate a variety of tests in research studies.

Brain imaging is integral to the diagnosis and prognosis of neurological disorders, offering valuable insights into patients’ cognitive status. Structural MRI correlates of neuropsychological and neuropsychiatric dysfunction in PD have been extensively investigated, revealing that structural brain alterations detectable by MRI are linked to specific cognitive deficits in PD. Research has identified volumetric reductions in limbic structures, particularly the hippocampus and amygdala, which correlate with verbal memory impairments in PD patients. Furthermore, patterns of cortical atrophy have been associated with other cognitive functions typically affected in the early stages of PD, indicating that structural changes extend beyond motor circuits to encompass broader cognitive networks [4,5,6,7,8,9,10]. However, there is limited evidence to suggest that preexisting brain damage predisposes PD patients to cognitive decline following DBS electrode implantation.

In a study by Planche et al., which examined brain structure volumes through MRI segmentation, it was observed that a reduced volume of the left nucleus accumbens may serve as a predictor for decline in specific cognitive domains in PD patients following DBS. Furthermore, the dimensions of other brain regions, including subcortical structure volumes and cortical thicknesses, were found to correlate with psychological test outcomes [11]. The reduction in volume of the nucleus basalis of Meynert has also been identified as a potential predictor of cognitive outcomes post-DBS. However, recent studies indicate that it accounts for only a minor portion of the variability in postoperative cognitive changes, necessitating further research [12]. Similarly, lower preoperative volumes of the hippocampus and thalamus have been associated with an increased risk of verbal memory decline following DBS, suggesting that structural MRI measures may assist in predicting susceptibility to cognitive deterioration in specific domains prior to surgery [6]. These findings imply that assessing brain atrophy can aid in evaluating a patient’s cognitive status and provide insights into the trajectory of change following an intervention.

Despite recent advancements, reliable predictors of cognitive decline following DBS that are applicable in clinical practice remain elusive [13]. Although several factors have been associated with an increased risk—such as older age at onset, lower levels of formal education, hippocampal atrophy, impaired baseline attention, lower performance on neurocognitive screening, higher levodopa equivalent doses, and the occurrence of serious adverse events—the evidence supporting these associations is inconsistent and relatively weak [13,14,15,16]. Given the challenges in identifying patients at higher risk of cognitive decline post-DBS implantation, there is a pressing need to identify predictors that can be routinely applied in clinical practice.

The objective of this study was to evaluate the correlation between preoperative brain morphometry and neuropsychological outcomes in a cohort of Parkinson’s disease patients undergoing deep brain stimulation in the subthalamic nucleus (DBS STN). The study aimed to identify potential prognostic markers of cognitive impairment following electrode implantation and to examine the impact of DBS on cognitive functions in these patients.

## 2. Methodology

### Study Design and Patient Selection

This research involved thirty-eight individuals diagnosed with PD who received DBS electrodes implanted in STN. Each participant had undergone neuropsychological evaluations both before and after the DBS procedure, and their preoperative MRI scans were accessible in the hospital’s database. The initial criteria for selecting PD patients for DBS STN were established in 1999 by the Core Assessment Program for Surgical Interventional Therapies in Parkinson’s Disease (CAPSIT-PD) group [17] and were subsequently revised [18]. The original CAPSIT-PD guidelines required patients to have a minimum disease duration of 5 years, at least 33% responsiveness to dopaminergic treatment, significant dyskinesia and fluctuations, no cognitive decline or severe depression, and no contraindications from neuroimaging or surgery.

All of study participants underwent one- or two-staged bilateral DBS STN under local anesthesia. Preoperative imaging comprised a 1.5 T MRI and stereotactic contrast-enhanced CT, employing Stereotactic Planning Software to ascertain the STN coordinates via both direct and indirect methodologies. Microelectrode recording (MER) was succeeded by macrostimulation, which was assessed by a neurophysiologist and a movement disorders neurologist using LeadPoint (Medtronic Inc., Minneapolis, MN, USA). A lateral control X-ray verified the electrode placement. The electrodes were secured at the burr holes with Stimlock, and the scalp incisions were sutured. Following the removal of the stereotactic frame, internal pulse generators (Activa SC, Medtronic, SC37603) were connected to the electrodes. Four weeks later, the stimulators were activated and adjusted to initiate stimulation without inducing adverse effects. If the stimulation effect was stable and balanced, medication was reduced.

Following initial data collection, we refined our analytical sample to enhance temporal homogeneity and address atypical cognitive trajectories. Four patients assessed >18 months post-operatively were excluded, as this extended interval substantially exceeds both the typical assessment window recommended for evaluating direct DBS effects and the median follow-up timing of the remaining cohort (12 months, IQR: 6–12 months). Additionally, three patients demonstrating reliable decline (RCI < −1.64) in ≥6 cognitive tests were excluded. This severity of decline substantially exceeded the cohort pattern (median: 1 test declined, IQR: 0–2). The final analytical sample comprised 31 patients with follow-up assessments conducted 3–18 months post-operatively (median: 12 months, IQR: 6–12 months).

All patients met standard DBS candidacy criteria, including: absence of cognitive decline or severe depression, disease duration ≥5 years, ≥33% response to levodopa challenge, and no contraindications from neuroimaging or comorbid conditions. None of the included patients met Movement Disorder Society criteria for Parkinson’s Disease–Mild Cognitive Impairment (PD-MCI) at baseline, based on comprehensive neuropsychological evaluation showing. Cognitive assessments were conducted as part of routine clinical protocol for all DBS candidates and follow-up patients.

## 3. Data Collection

Demographic and clinical data, including age, gender, prescribed medications, clinical assessment, and disease duration, were collected from the hospital database. Clinical and neuropsychological assessments were conducted both before and 3–24 months after the surgery. Neuropsychological tests before the surgery aimed at evaluating cognitive functions, while postoperative tests were conducted to assess cognitive function after implantation. Standardized tests including Mini Mental State Examination (MMSE), Addenbrooke’s Cognitive Examination III (ACEIII), Montreal Cognitive Assessment (MOCA), Executive Clock Drawing Task (CLOX), Auditory Verbal Learning Test (AVLT), Beck Depression Inventory (BDI), Benton’s Judgment of Line Orientation (BentonJLO), Boston Naming Test (BNT), Trail Making Test (TMT), Verbal Fluency Test, Wechsler Adult Intelligence Scale (WAIS-R), and Tower of London (TOL) were used for both pre- and postoperative evaluations.

Tests were divided into categories of cognitive domains they assess: (1) global cognitive functioning (MMSE, ACEIII, MOCA), (2) visuospatial functions (CLOX, Benton JLO), (3) memory functions (AVLT), (4) language functions (BNT, Verbal Fluency Test), (5) attention and processing speed (TMT-A, WAIS-R digit span), (6) executive functions (TMT-B, Tower of London, WAIS-R similarities), and (7) mood and affective functions (BDI), consistent with established neuropsychological classification frameworks [19].

Parameters, including dimensions and intensities of specific brain structures related to cognitive functions and Parkinson’s disease, were chosen based on literature review and the pathophysiology of Parkinson’s disease. Selected forty-two parameters included bilateral measurements of precentral gyrus, middle frontal gyrus, superior frontal gyrus, superior temporal gyrus, lateral ventricle, lentiform nucleus, caudate nucleus head, insula, thalamus, and substantia nigra and unilateral (midline) measurements of midbrain, cingulate gyrus, pons, cerebellar vermis, and hippocampal cortex. Manual measurements of selected parameters on MRI images were performed using a ruler and intensity meter [4,11,20,21]. Parkinsonian symptoms were assessed by movement disorders specialist using the Unified Parkinson’s Disease Rating Scale (UPDRS). Conversion factors of antiparkinsonian drugs were used to calculate Cumulative L-Dopa equivalent daily dose (LEDD) [22].

## 4. Statistical Analysis

Statistical analyses were performed using Python 3.8. The distribution of all continuous data was evaluated with the Shapiro–Wilk test. Given that the data did not satisfy the assumptions for parametric tests, group-level cognitive change was evaluated using Wilcoxon tests comparing pre- and post-operative scores. Data are presented as median [interquartile range], given non-normal distributions. Effect sizes were calculated as Cohen’s d using pooled standard deviations, with interpretation: small (0.2 ≤ |d| < 0.5), medium (0.5 ≤ |d| < 0.8), and large (|d| ≥ 0.8).

For the analysis of changes in neuropsychological test results across different time points for each patient with available data for the specific test, the significance of these changes was assessed using the Reliable Change Index (RCI). The RCI was calculated using the formula RCI = (test score at follow-up—test score at baseline)/SDdiff, where SDdiff denotes the standard error of the difference score. Spearman’s correlation coefficients were utilized to compute correlations between baseline MRI parameters and cognitive change (RCI scores). These correlations were visualized using a heatmap. Given the large number of comparisons, we applied false discovery rate (FDR) correction. No correlations survived FDR correction (q < 0.05), which is expected given the modest sample size (*n* = 30) and exploratory nature of this hypothesis-generating study. For transparency and to facilitate future meta-analyses, we report all correlations with uncorrected *p* < 0.05, acknowledging the elevated risk of false positives.

## 5. Results

A cohort of 31 patients diagnosed with Parkinson’s disease (PD) and undergoing deep brain stimulation (DBS) therapy was included in this study following sample refinement. Comprehensive data were collected, including demographic information, clinical assessments, pre-operative brain MRI, and both pre- and post-operative neuropsychological evaluations. The demographic and clinical characteristics of the patients are detailed in Table 1. The median age of the participants was 54 years (interquartile range [IQR] 48–62), with a median disease duration of 8,5 years (IQR 7–10). The median follow-up interval between surgery and post-operative assessment was 12 months (IQR 6–12 months). Notable differences were observed in postoperative clinical outcomes, evidenced by an increase in Unified Parkinson’s Disease Rating Scale (UPDRS) OFF scores (*p* < 0.001) and a reduction in levodopa equivalent daily dose (LEDD) (*p* < 0.001). In terms of neuropsychological assessments (refer to Table 2), postoperative patients demonstrated significantly poorer performance on the Boston Naming Test (BNT) (*p* = 0.041), and all subtests of the Verbal Fluency Test (letter K: *p* = 0.004, d = −0.64; letter P: *p* = 0.016, d = −0.49; animals: *p* < 0.001, d = −0.84; fruits/vegetables: *p* = 0.027, d = −0.50). Other cognitive domains showed no significant group-level changes.

**Figure 1:**
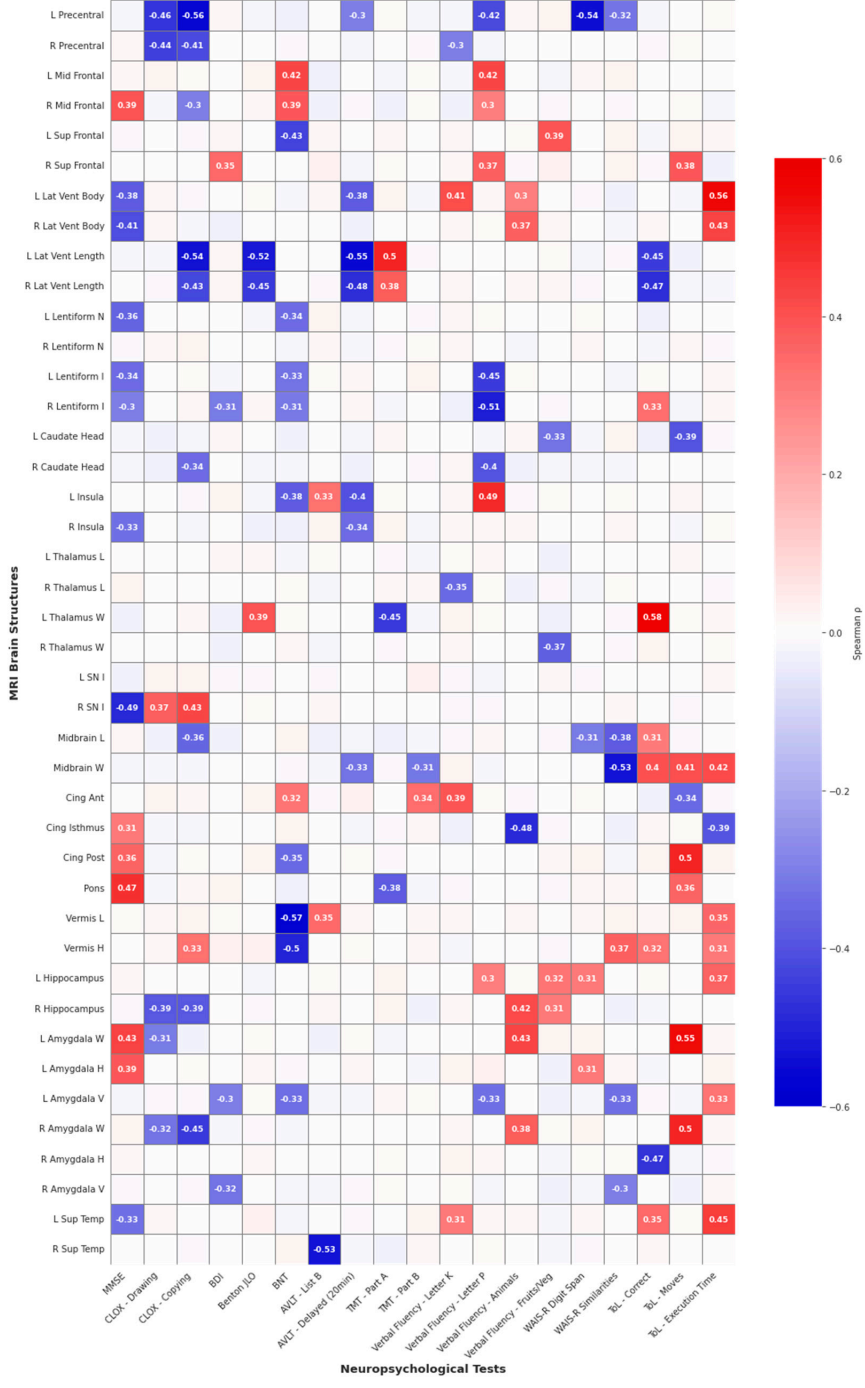
Heatmap of the correlations between neuropsychological test results and MRI parameters (correlation coefficient |r| > 0.3). Abbreviations: L/R = left/right; Lat Vent = lateral ventricle; W/L/H = width/length/height; I = intensity; N = nucleus; Cing = cingulate (Ant/Post = anterior/posterior); SN = substantia nigra; Sup/Mid/Temp = superior/middle/ temporal.

Spearman correlation analyses identified significant associations between MRI-derived brain structural measures and longitudinal changes in neuropsychological performance (Table 3 and Figure 1), with correlations ranging from moderate to strong (ρ = −0.57 to +0.58, *p* = 0.002 to *p* = 0.024). The most pronounced effects were observed in executive functioning, where the CLOX copying task exhibited the strongest negative correlation with left precentral gyrus width (ρ = −0.56, *p* = 0.006), while larger bilateral amygdala and left thalamus dimensions were associated with better performance in the ToL test (left thalamus—correct responses: ρ = +0.58, *p* = 0.002; left amygdala—total moves: ρ = +0.55, *p* = 0.005). Memory functions demonstrated significant negative correlations, with AVLT delayed recall negatively correlated with bilateral lateral ventricular enlargement (ρ = −0.55, *p* = 0.003), AVLT list B most strongly associated with right superior temporal gyrus width (ρ = −0.53, *p* = 0.005), and WAIS-R Digit Span negatively correlated with left precentral gyrus width (ρ = −0.54, *p* = 0.005). Language abilities revealed a decline in the BNT associated with larger cerebellar vermis (ρ = −0.57, *p* = 0.005), while semantic fluency tasks showed positive correlations with bilateral hippocampal and amygdala dimensions (left amygdala: ρ = +0.43, *p* = 0.023). Global cognitive screening (MMSE) was negatively correlated with right substantia nigra intensity (ρ = −0.49, *p* = 0.023). Overall, larger bilateral lateral ventricles and left precentral gyrus, along with smaller dimensions of limbic structures (amygdala, hippocampus) and brainstem regions, consistently emerged as associated with poorer cognitive outcomes following DBS.

## 6. Discussion

The present study provides novel insights into the association between preoperative brain morphology and postoperative cognitive alterations in patients with Parkinson’s disease (PD) undergoing bilateral deep brain stimulation of the subthalamic nucleus (STN-DBS). Although the primary therapeutic objective of DBS is to alleviate motor symptoms and enhance quality of life, accumulating evidence indicates that the intervention may also induce selective cognitive side effects in a subset of patients [3,13,15,23,24]. Our findings support previous reports suggesting that STN-DBS does not typically result in a generalized cognitive decline but rather leads to domain-specific changes, most consistently observed in verbal fluency (phonemic and semantic), with additional decline in confrontation naming (Boston Naming Test).

### 6.1. Patterns of Cognitive Change After STN-DBS

In our cohort, we observed significant postoperative declines primarily in language domains: verbal fluency (phonemic and semantic) and confrontation naming (BNT). Effect sizes were largest for semantic fluency (animals: Cohen’s d = −0.84, large effect) and phonemic fluency (letter K: d = −0.64, medium effect). In contrast, global cognition (MMSE), memory (AVLT), executive functions (WAIS-R Similarities, Tower of London), attention (TMT), visuospatial abilities (Benton JLO), and mood (BDI) showed no significant group-level changes. This cognitive profile aligns with previous studies indicating that STN-DBS selectively impairs verbal fluency while largely preserving other cognitive domains [3,6,15,23]. The decline in verbal fluency, documented across all subtests in our study, is the most consistently observed cognitive effect of STN-DBS, supported by robust evidence from randomized trials attributing this change to the surgical intervention itself rather than disease progression [3]. Mechanistically, this deficit has been associated with transient or chronic disruption of frontostriatal circuits, particularly involving the dorsolateral prefrontal cortex, either due to electrode trajectory through caudate and frontal white matter or from stimulation-induced modulation of non-motor STN territories.

Notably, the absence of significant memory decline and preserved global cognition in our sample may reflect our exclusion of patients with atypical cognitive trajectories (≥6 tests with reliable decline) and extended follow-up intervals (>18 months), which likely removed cases where disease progression or surgical complications contributed to broader impairment. Our findings suggest that uncomplicated STN-DBS primarily correlates with language/fluency networks, with relative sparing of memory and global cognition in the majority of patients within the first 18 months post-operatively.

### 6.2. Morphometric Correlates of Postoperative Cognitive Outcome

A significant contribution of this study is the identification of straightforward MRI-based, clinically accessible MRI morphometric markers associated with cognitive outcomes following STN-DBS. Notably, increased lateral ventricular dimensions were consistently correlated with diminished postoperative performance across various domains, including memory, visuospatial abilities, and overall cognitive function. This observation corroborates the findings of Bourne et al. [25], who proposed ventricular width as an indicator of limited structural reserve and an elevated risk of postoperative complications. Ventricular enlargement may reflect widespread cortical and subcortical atrophy, potentially diminishing the brain’s capacity to compensate for disruptions caused by surgery or stimulation.

The left-lateralized associations observed in our data are consistent with earlier reports of hemispheric asymmetry in PD-related neurodegeneration [26], possibly reflecting a left-dominant vulnerability of language and verbal memory networks.

Our study identified cortical morphometric factors, such as an expanded base of the precentral gyrus and diminished dimensions of the cingulate gyrus, that correlated with diminished executive cognitive performance. The cingulate cortex is integral to executive control, attention, and error monitoring, and atrophy in this region has been linked to executive dysfunction in PD. These findings extend the focus of previous imaging studies which have concentrated on cortical thickness and white matter integrity [3,6,15,27,28,29], by demonstrating that easily obtainable linear measurements from standard MRI scans can provide clinically relevant prognostic information. The current findings resonate with and expand upon prior work by Planche et al. [11] and Kübler et al. [12], who reported that volumes of subcortical structures such as the nucleus accumbens and nucleus basalis of Meynert correlate with cognitive outcomes following STN-DBS. Although we did not directly assess these nuclei, our results implicate other limbic and associative structures within the broader fronto-limbic network. Similarly, studies linking thalamic and hippocampal volumes to postoperative verbal memory [6] align with our observed correlations between limbic volumes and language performance. The consistency of negative correlations between ventricular size and cognition, alongside correlations between limbic structures and performance, suggests that both global and regional markers of neurodegeneration contribute to postoperative vulnerability. It is important to note that while we report correlations with uncorrected *p* < 0.05, most did not survive false discovery rate correction (q < 0.05), reflecting the exploratory nature of this hypothesis-generating study. The consistency of findings—with bilateral effects for several structures (lateral ventricles, amygdala) and convergent associations across related cognitive measures (multiple fluency subtests, executive tasks)—strengthens confidence beyond individual statistical thresholds. Nevertheless, these associations should be interpreted cautiously as preliminary markers requiring independent validation.

### 6.3. Possible Mechanisms Underlying Cognitive Vulnerability

The mechanisms linking brain morphology to cognitive outcomes following STN-DBS are likely multifactorial. Ventricular enlargement may signify a widespread loss of both gray and white matter, thereby reducing the redundancy of cognitive networks. Limbic atrophy could impair declarative memory and emotional regulation, while alterations in cortical folding might affect connectivity and synaptic efficiency in association areas critical for executive and language processing. From a procedural perspective, factors such as electrode placement within or near associative and limbic STN subterritories, microlesional effects from insertion, and current spread during stimulation may interact with preexisting structural deficits, potentially exacerbating cognitive vulnerability. Additionally, postoperative scarring and gliosis could further contribute to network disruption.

### 6.4. Clinical Implications for DBS Candidacy and Counseling

Identifying patients at an elevated risk for cognitive decline following deep brain stimulation (DBS) holds significant implications for surgical decision-making and patient counseling. In instances where structural MRI reveals pronounced ventricular enlargement, limbic atrophy, or cortical alterations indicative of diminished reserve, alternative targets such as the globus pallidus internus (GPi) may be more suitable [24], given that GPi-DBS may present a more favorable cognitive profile. Furthermore, patients with heightened structural risk could benefit from enhanced postoperative cognitive monitoring, early initiation of cognitive rehabilitation, and tailored stimulation programming to mitigate cognitive side effects. Notably, our findings suggest that relatively straightforward morphometric measurements could be incorporated into routine preoperative imaging assessments. Standardizing these measures across centers would facilitate risk stratification and enable prospective validation in larger cohorts.

### 6.5. Motor Outcomes and Their Relation to Cognition

As anticipated, our cohort exhibited substantial postoperative reductions in the levodopa equivalent daily dose (LEDD) and improvements in motor scores in the OFF state, aligning with the established motor efficacy of STN-DBS. However, motor scores in the ON state did not demonstrate significant changes, a pattern that may indicate ceiling effects or preoperative optimization of dopaminergic therapy. The observed dissociation between motor and cognitive outcomes underscores the necessity of considering non-motor endpoints in the evaluation of DBS outcomes.

### 6.6. Limitations

This study acknowledges some limitations. The relatively small, single-center sample may constrain the generalizability of the findings and elevate the risk of selection bias. The study design restricted the neuropsychological battery to clinically indicated tests, thereby limiting a more comprehensive assessment of domains such as visuoconstruction or social cognition. The follow-up period, spanning 3 to 18 months, may not sufficiently capture late-emerging cognitive changes.

We employed manual linear MRI measurements rather than automated volumetric segmentation. While manual measurement is inherently more variable than automated methods, it offers satisfactory performance on lower-resolution clinical scans, allows quality control for motion artifacts, atypical anatomy, and pathological features (e.g., surgical clips) that can cause automated segmentation failures. Finally, linear measurements are clinically accessible and can be readily implemented in routine preoperative assessments without specialized software or expertise, enhancing translational potential. Nevertheless, future prospective studies employing research-grade MRI with standardized acquisition protocols would enable automated volumetric analysis, enhancing precision, and providing comprehensive whole-brain morphometry beyond the structures we assessed.

Lastly, the absence of correction for multiple comparisons suggests that some findings may represent type I error, underscoring the necessity for replication. The anatomical consistency of findings—with bilateral effects and convergent associations across related cognitive measures—provides some confidence, but independent replication in larger cohorts is essential before these morphometric markers can guide clinical decision-making.

### 6.7. Future Directions

Future research should prioritize prospective, multicenter studies employing standardized morphometric protocols and extended follow-up periods to capture long-term cognitive trajectories. The combination of simple linear measures with advanced volumetric, cortical thickness, and diffusion tensor imaging analyses may enhance predictive accuracy. Integrating imaging data with baseline neuropsychological performance, genetic factors, and intraoperative electrophysiology could yield comprehensive prognostic models. Incorporating structural brain evaluations into pre-surgery assessments can help customize DBS approaches, enhance target selection, and improve patient outcomes by balancing motor improvements with potential cognitive risks.

## 7. Conclusions

In conclusion, our study confirms that patients with Parkinson’s disease (PD) who have undergone subthalamic nucleus deep brain stimulation (STN-DBS) exhibit specific cognitive changes, particularly in verbal fluency, while maintaining other cognitive functions such as attention and working memory. We demonstrate that simple morphometric MRI measures, including ventricular dimensions, limbic structure size, and cortical gyral parameters, are associated with postoperative cognitive trajectories. These easily accessible markers may serve as valuable tools for hypothesis generation in preoperative risk assessment, pending validation in prospective studies. By integrating structural brain evaluations into presurgical planning, future approaches may achieve more personalized DBS strategies that optimize motor benefits while minimizing cognitive risks.

## Figures and Tables

**Table 1:** Demographic and clinical characteristics of the patients.

Variables	Pre-DBS Median [Q1, Q3]	Post-DBS Median [Q1, Q3]	*p* Value
Age	54 [48–62]		
Gender (male/female)	21/10		
PD duration (years)	8.5 [7–10]		
Follow-up interval (months)		12 [6–12]	
DBS one staged/two-staged bilateral procedure	24/7		
UPDRS III ON	6.0 [5.0, 9.5]	5.0 [4.0, 7.0]	0.159
UPDRS III OFF	35.0 [32.0, 43.5]	37.0 [34.0, 46.0]	<0.001 *
LEDD	1430.0 [1018.8, 1907.5]	752.5 [505.0, 1131.2]	<0.001 *

* means it is statistically significant.

**Table 2:** Neuropsychological testing scores before and after DBS implantation.

Variable	*n*	Pre-DBS Median [Q1, Q3]	Post-DBS Median [Q1, Q3]	*p* Value	Cohen’s d	Effect Size
MMSE	21	28.0 [28.0, 29.0]	28.0 [27.0, 29.0]	0.170	−0.338	Small
CLOX—Drawing	22	13.0 [11.5, 14.0]	12.5 [12.0, 13.0]	0.170	−0.336	Small
CLOX—Copying	22	14.0 [13.2, 15.0]	14.0 [14.0, 14.8]	0.805	0.046	-
BDI	28	12.5 [5.5, 18.2]	9.5 [5.5, 13.2]	0.186	−0.295	Small
Benton JLO	25	24.0 [21.0, 28.0]	25.0 [20.0, 29.0]	0.484	−0.159	-
BNT	22	14.0 [13.0, 15.0]	14.0 [12.2, 14.8]	0.041 *	−0.469	Small
AVLT—Total (A/B)	27	40.0 [34.5, 44.0]	35.0 [31.5, 42.5]	0.116	−0.302	Small
AVLT—A after 20 min	27	7.0 [4.5, 8.5]	5.0 [4.0, 7.0]	0.088	−0.330	Small
TMT—Part A	25	35.0 [30.0, 55.0]	38.0 [29.0, 49.0]	0.864	−0.015	-
TMT—Part B	25	91.0 [78.0, 140.0]	105.0 [72.0, 140.0]	0.637	0.217	Small
Verbal Fluency—Letter K	26	17.0 [14.2, 19.0]	14.0 [11.2, 16.8]	0.004 *	−0.643	Medium
Verbal Fluency—Letter P	26	14.0 [12.0, 15.0]	13.0 [10.2, 15.5]	0.016 *	−0.490	Small
Verbal Fluency—Animals	28	20.0 [15.8, 23.2]	17.5 [13.8, 19.0]	<0.001 *	−0.839	Large
Verbal Fluency—Fruits/Vegetables	25	19.0 [16.0, 21.0]	17.0 [13.0, 20.0]	0.027 *	−0.501	Medium
WAIS-R—Digit Span	25	10.0 [8.0, 12.0]	9.0 [9.0, 11.0]	0.277	−0.222	Small
WAIS-R—Similarities	25	17.0 [13.0, 20.0]	15.0 [13.0, 20.0]	0.106	−0.365	Small
ToL—Correct Moves	25	3.0 [2.0, 5.0]	3.0 [2.0, 5.0]	0.447	−0.190	-
ToL—Total Moves	24	35.5 [22.2, 48.2]	32.0 [21.8, 44.2]	0.781	0.071	-
ToL—Execution Time	23	264.0 [186.0, 370.0]	301.0 [196.0, 368.0]	0.602	−0.022	-

Note: Data presented as median [interquartile range]. Effect size interpretation: Small (0.2 ≤ |d| < 0.5), Medium (0.5 ≤ |d| < 0.8), Large (|d| ≥ 0.8). * *p* < 0.05.

**Table 3:** Significant correlations (*p* < 0.05) of the neuropsychological tests results with MRI parameters and demographical and clinical data. A negative correlation means a higher risk of decline with an increase in each parameter.

Test	Vulnerable Structures (Negative Correlations)	Protective Structures (Positive Correlations)	Strongest Effect
**Executive Functions**			
CLOX—Drawing	L/R Precentral gyrus	—	ρ = −0.46, *p* = 0.024
CLOX—Copy	L Precentral gyrus, L/R Lateral ventricles (length), R Amygdala	R Substantia nigra	ρ = −0.56, *p* = 0.006
ToL—Correct responses	L/R Lateral ventricles (length), R Amygdala (height)	L Thalamus, Midbrain	ρ = +0.58, *p* = 0.002
ToL—Moves	—	L/R Amygdala, Posterior cingulate, Midbrain	ρ = +0.55, *p* = 0.005
ToL—Total time	—	L/R Lateral ventricles (width), Midbrain, L Superior temporal	ρ = +0.56, *p* = 0.004
Phonemic Fluency (Verbal Fluency: Letter P)	L Precentral gyrus, L/R Lentiform, R Caudate	L Middle frontal, L Insula, L Lateral ventricle (width), Anterior cingulate	ρ = −0.51, *p* = 0.008
**Memory Functions**			
AVLT—20 min Delayed	L/R Lateral ventricles (length), L Insula	—	ρ = −0.55, *p* = 0.003
AVLT—List B	R Superior temporal	—	ρ = −0.53, *p* = 0.005
WAIS-R Digit Span	L Precentral gyrus	—	ρ = −0.54, *p* = 0.005
**Attention and Processing Speed**			
TMT—Part A	L Thalamus	L Lateral ventricle (length)	ρ = −0.45, *p* = 0.023
**Visuospatial Functions**			
Benton JLO	L/R Lateral ventricles (length)	—	ρ = −0.52, *p* = 0.008
**Language Functions**			
Boston Naming Test	Cerebellar vermis (length and height), L Superior frontal	L Middle frontal	ρ = −0.57, *p* = 0.005
Animal Naming	Cingulate isthmus	L/R Hippocampus, L/R Amygdala	ρ = +0.43, *p* = 0.023
Fruit/Vegetable Naming	—	—	—
**Global Cognitive Screening**			
MMSE	R Substantia nigra	Pons	ρ = −0.49, *p* = 0.023
**Abstract Reasoning**			
WAIS-R Similarities	Midbrain	—	ρ = −0.53, *p* = 0.007
**Affective/Emotional Functions**			
BDI	—	—	—

Abbreviations: AVLT = Auditory Verbal Learning Test; CLOX = Clock Drawing Test; JLO = Judgment of Line Orientation; MMSE = Mini-Mental State Examination; TMT-A = Trail Making Test Part A; ToL = Tower of London; WAIS-R = Wechsler Adult Intelligence Scale-Revised. Note: Negative correlations indicate that larger brain volumes/metrics are associated with greater cognitive decline after DBS. Positive correlations indicate that larger brain volumes/metrics are associated with better cognitive outcomes or less decline after DBS.

## Data Availability

The original contributions presented in this study are included in the article/supplementary material. Further inquiries can be directed to the corresponding author.

## Supplementary Materials

The following supporting information can be downloaded at: https://www.mdpi.com/article/10.3390/jcm14238414/s1, Table S1: Measurements of selected parameters on brain MRIs.

## References

[1] Rothlind J.C., York M.K., Carlson K., Luo P., Marks W.J., Weaver F.M., Stern M., Follett K., Reda D., CSP-468 Study Group (2015). Neuropsychological changes following deep brain stimulation surgery for Parkinson’s disease: Comparisons of treatment at pallidal and subthalamic targets versus best medical therapy. J. Neurol. Neurosurg. Psychiatry.

[2] Rughani A., Schwalb J.M., Sidiropoulos C., Pilitsis J., Ramirez-Zamora A., Sweet J.A., Mittal S., Espay A.J., Martinez J.G., Abosch A. (2018). Congress of Neurological Surgeons Systematic Review and Evidence-Based Guideline on Subthalamic Nucleus and Globus Pallidus Internus Deep Brain Stimulation for the Treatment of Patients with Parkinson’s Disease: Executive Summary. Neurosurgery.

[3] Witt K., Daniels C., Reiff J., Krack P., Volkmann J., Pinsker M.O., Krause M., Tronnier V., Kloss M., Schnitzler A. (2008). Neuropsychological and psychiatric changes after deep brain stimulation for Parkinson’s disease: A randomised, multicentre study. Lancet Neurol..

[4] Pagonabarraga J., Corcuera-Solano I., Vives-Gilabert Y., Llebaria G., García-Sánchez C., Pascual-Sedano B., Delfino M., Kulisevsky J., Gómez-Ansón B. (2013). Pattern of regional cortical thinning associated with cognitive deterioration in Parkinson’s disease. PLoS ONE.

[5] Filippi M., Canu E., Donzuso G., Stojkovic T., Basaia S., Stankovic I., Tomic A., Markovic V., Petrovic I., Stefanova E. (2020). Tracking Cortical Changes Throughout Cognitive Decline in Parkinson’s Disease. Mov. Disord..

[6] Geevarghese R., Lumsden D.E., Costello A., Hulse N., Ayis S., Samuel M., Ashkan K. (2016). Verbal Memory Decline following DBS for Parkinson’s Disease: Structural Volumetric MRI Relationships. PLoS ONE.

[7] Filoteo J.V., Reed J.D., Litvan I., Harrington D.L. (2014). Volumetric correlates of cognitive functioning in nondemented patients with Parkinson’s disease. Mov. Disord..

[8] Alzahrani H., Venneri A. (2015). Cognitive and neuroanatomical correlates of neuropsychiatric symptoms in Parkinson’s disease: A systematic review. J. Neurol. Sci..

[9] Pereira J.B., Junqué C., Martí M.J., Ramirez-Ruiz B., Bartrés-Faz D., Tolosa E. (2009). Structural brain correlates of verbal fluency in Parkinson’s disease. NeuroReport.

[10] Ibarretxe-Bilbao N., Junque C., Marti M.J., Tolosa E. (2011). Brain structural MRI correlates of cognitive dysfunctions in Parkinson’s disease. J. Neurol. Sci..

[11] Planche V., Munsch F., Pereira B., de Schlichting E., Vidal T., Coste J., Morand D., de Chazeron D., Derost P., Debilly B. (2018). Anatomical predictors of cognitive decline after subthalamic stimulation in Parkinson’s disease. Brain Struct. Funct..

[12] Kübler D., Wellmann S.K., Kaminski J., Skowronek C., Schneider G.H., Neumann W.J., Ritter K., Kühn A. (2022). Nucleus basalis of Meynert predicts cognition after deep brain stimulation in Parkinson’s disease. Park. Relat Disord.

[13] Massano J., Garrett C. (2012). Deep brain stimulation and cognitive decline in Parkinson’s disease: A clinical review. Front. Neurol..

[14] Heo J.H., Lee K.M., Paek S.H., Kim M.J., Lee J.Y., Kim J.Y., Cho S.Y., Lim Y.H., Kim M.R., Jeong S.Y. (2008). The effects of bilateral subthalamic nucleus deep brain stimulation (STN DBS) on cognition in Parkinson disease. J. Neurol. Sci..

[15] Smeding H.M., Speelman J.D., Huizenga H.M., Schuurman P.R., Schmand B. (2011). Predictors of cognitive and psychosocial outcome after STN DBS in Parkinson’s Disease. J. Neurol. Neurosurg. Psychiatry.

[16] Rothlind J.C., York M.K., Luo P., Carlson K., Marks W.J., Weaver F.M., Stern M., Follett K.A., Duda J.E., Reda D.J. (2022). Predictors of multi-domain cognitive decline following DBS for treatment of Parkinson’s disease. Park. Relat. Disord..

[17] Defer G.L., Widner H., Marie R.M., Remy P., Levivier M. (1999). Core assessment program for surgical interventional therapies in Parkinson’s disease (CAPSIT-PD). Mov. Disord..

[18] Deuschl G., Antonini A., Costa J., Śmiłowska K., Berg D., Corvol J.C., Fabbrini G., Ferreira J., Foltynie T., Mir P. (2022). European Academy of Neurology/Movement Disorder Society-European Section Guideline on the Treatment of Parkinson’s Disease: I. Invasive Therapies. Mov. Disord..

[19] Romann A.J., Dornelles S., Maineri N.L., Rieder C.R.M., Olchik M.R. (2012). Cognitive assessment instruments in Parkinson’s disease patients undergoing deep brain stimulation. Dement. Neuropsychol..

[20] Biundo R., Calabrese M., Weis L., Facchini S., Ricchieri G., Gallo P., Antonini A. (2013). Anatomical correlates of cognitive functions in early Parkinson’s disease patients. PLoS ONE.

[21] Zeighami Y., Ulla M., Iturria-Medina Y., Dadar M., Zhang Y., Larcher K.M., Fonov V., Evans A.C., Collins D.L., Dagher A. (2015). Network structure of brain atrophy in de novo Parkinson’s disease. eLife.

[22] Jost S.T., Kaldenbach M.A., Antonini A., Martinez-Martin P., Timmermann L., Odin P., Katzenschlager R., Borgohain R., Fasano A., Stocchi F. (2023). Levodopa Dose Equivalency in Parkinson’s Disease: Updated Systematic Review and Proposals. Mov. Disord..

[23] Maheshwary A., Mohite D., Omole J.A., Bhatti K.S., Khan S. (2020). Is Deep Brain Stimulation Associated with Detrimental Effects on Cognitive Functions in Patients of Parkinson’s Disease? A Systematic Review. Cureus.

[24] Combs H.L., Folley B.S., Berry D.T., Segerstrom S.C., Han D.Y., Anderson-Mooney A.J., Walls B.D., van Horne C. (2015). Cognition and Depression Following Deep Brain Stimulation of the Subthalamic Nucleus and Globus Pallidus Pars Internus in Parkinson’s Disease: A Meta-Analysis. Neuropsychol. Rev..

[25] Bourne S.K., Conrad A., Konrad P.E., Neimat J.S., Davis T.L. (2012). Ventricular width and complicated recovery following deep brain stimulation surgery. Stereotact. Funct. Neurosurg..

[26] Voruz P., Haegelen C., Assal F., Drapier S., Drapier D., Sauleau P., Vérin M., Péron J.A. (2023). Motor Symptom Asymmetry Predicts Cognitive and Neuropsychiatric Profile Following Deep Brain Stimulation of the Subthalamic Nucleus in Parkinson’s Disease: A 5-Year Longitudinal Study. Arch. Clin. Neuropsychol..

[27] Radziunas A., Deltuva V.P., Tamasauskas A., Gleizniene R., Pranckeviciene A., Surkiene D., Bunevicius A. (2018). Neuropsychiatric complications and neuroimaging characteristics after deep brain stimulation surgery for Parkinson’s disease. Brain Imaging Behav..

[28] Hanganu A., Bedetti C., Degroot C., Mejia-Constain B., Lafontaine A.-L., Soland V., Chouinard S., Bruneau M.-A., Mellah S., Belleville S. (2014). Mild cognitive impairment is linked with faster rate of cortical thinning in patients with Parkinson’s disease longitudinally. Brain.

[29] Wang F., Lai Y., Pan Y., Li H., Liu Q., Sun B. (2022). A systematic review of brain morphometry related to deep brain stimulation outcome in Parkinson’s disease. NPJ Park. Dis..

