# Supplementary material for: Association of Preoperative Linear MRI Measures with Domain-Specific Cognitive Change After Subthalamic Nucleus Deep Brain Stimulation in Parkinson’s Disease
Source: J Clin Med. 2025 Nov 27;14(23):8414. doi: 10.3390/jcm14238414 (PMC12692749; doi:10.3390/jcm14238414)
Supplement: Supplementary file 1 [file jcm-14-08414-s001.zip › jcm-3946241-supplementary.pdf]

Supplementary Table S1. Measurements of selected parameters on brain MRIs.

Structure	Measurement/Dimention	Plane
	Bilateral	
Precentral gyrus	lenght	transverse
Middle frontal gyrus	lenght	
Superior frontal gyrus	lenght	
Lateral ventricle	Lenght, trunk	
Lentiform n.	Lenght, intensity	
Caudate n. head	lenght	
Insula	lenght	
Thalamus	Lenght, width	
Substantia nigra	intensity	
Superior temporal gyrus	width	frontal
	Midline	
Midbrain	Lenght, width	transverse
Cingulate gyrus	anterior, isthmus, posterior	sagittal
Pons	lenght	
Cerebellar vermis	Lenght, height	
Hippocampal cortex	Width	frontal
Amygdala	Lenght, width, intensity	
